# Concurrent Kaposi Sarcoma and B-cell Lymphoma in an HIV-Negative Patient: A Case Report

**DOI:** 10.7759/cureus.113592

**Published:** 2026-07-29

**Authors:** Marwa Faik Ouahab, Bouchra El Ghouti, Soumiya Chiheb, Madiha Eljazouly

**Affiliations:** 1 Dermatology, Cheikh Khalifa International University Hospital, Mohammed VI University of Health Sciences, Casablanca, MAR

**Keywords:** b-cell lymphocytic lymphoma, cll, diagnostic delay, hhv-8, hiv negative kaposi sarcoma, kaposi's sarcoma, therapeutic escape

## Abstract

We report the case of a 70-year-old non-immunocompromised male patient with coexisting classic Kaposi sarcoma (KS) and stage IV B-cell lymphocytic lymphoma. KS had been evolving insidiously since 2017 but was only confirmed in 2024 following a retrospective histological re-review. The patient received two lines of systemic chemotherapy, rituximab plus bendamustine (R-BENDA) followed by paclitaxel, with transient clinical responses before disease progression. This case suggests that the intrinsic immune dysfunction associated with chronic lymphocytic leukemia (CLL) may provide a permissive environment for human herpesvirus 8 (HHV-8) reactivation in the absence of classically recognized immunodeficiency states (HIV infection, iatrogenic immunosuppression, or primary immunodeficiency) and underscores the importance of histological re-review of atypical or long-standing cutaneous lesions.

## Introduction

Kaposi sarcoma (KS) is a low-grade vascular neoplasm of endothelial origin associated with human herpesvirus 8 (HHV-8). It is classically classified into four epidemiologic subtypes: epidemic KS, associated with HIV/AIDS; iatrogenic KS, occurring in the setting of immunosuppression, particularly after solid organ transplantation; endemic KS, observed primarily in certain regions of sub-Saharan Africa; and classic KS [1]. The classic form typically affects immunocompetent elderly men of Mediterranean or Eastern European descent and is characterized by a chronic, indolent course [2]. More recently, a fifth epidemiologic variant has been increasingly recognized: non-epidemic KS occurring in HIV-negative men who have sex with men (MSM) in the absence of any other identifiable cause of immunodeficiency [3].

B-cell lymphocytic lymphoma, also known as small lymphocytic lymphoma/chronic lymphocytic leukemia (SLL/CLL) is a recognized cause of intrinsic functional immunosuppression, even in the absence of cytotoxic therapy, driven in part by impaired T-cell and natural killer (NK)-cell surveillance. It has been associated with increased susceptibility to herpesvirus reactivation and secondary malignancies [4]. Although the coexistence of KS with hematologic or lymphoid neoplasms has previously been documented, the concurrent occurrence of KS and CLL/SLL in an HIV-negative, non-transplant patient represents an uncommon clinical association.

We report a case of concurrent KS and CLL/SLL in a 70-year-old HIV-negative, non-transplant patient without prior iatrogenic immunosuppression, in whom cutaneous KS lesions had been misdiagnosed for seven years before histopathological confirmation.

## Case presentation

A 70-year-old male patient with a medical history of chronic gastroesophageal reflux disease presented with violaceous nodules on the right foot, including the plantar surface, that had evolved over eight years. He had no known immunodeficiency and no history of long-term corticosteroid use.

In 2017, he developed erythematous lesions associated with edema on the right foot. Initial investigations, including a skin biopsy, were inconclusive, and management was limited to conservative lifestyle measures.

A complete blood count performed in 2019 incidentally showed leukocytosis (12 × 10⁹/L) with absolute lymphocytosis (8.227 × 10⁹/L). This finding was attributed at the time to a presumed infectious process, and no further hematological work-up, including flow cytometry, was performed.

In November 2022, evaluation for persistent fatigue incidentally revealed leukocytosis and lymphocytosis. A bone marrow biopsy demonstrated diffuse infiltration of the bone marrow by small lymphoid cells. Immunohistochemistry showed diffuse expression of CD20, CD5, and CD23 by the neoplastic cells, whereas cyclin D1 and CD10 were negative; CD3 highlighted only reactive T lymphocytes. These morphological and immunophenotypic findings were consistent with stage IV B-cell CLL/SLL. No molecular studies were available. Baseline lactate dehydrogenase and beta-2 microglobulin levels were within normal limits. As none of the treatment criteria specified in the International Workshop on Chronic Lymphocytic Leukemia (iwCLL) guidelines were met, a watch-and-wait strategy with quarterly blood count monitoring was adopted. During follow-up from 2022 to 2024, the absolute lymphocyte count gradually increased from approximately 8 × 10⁹/L to 12 × 10⁹/L, without significant hematological progression.

In July 2024, new nodular lesions developed on the right foot. Clinical examination revealed extensive confluent hyperkeratotic plaques and verrucous nodules covering the plantar, lateral, and dorsal surfaces, with overlying keratotic crusting, areas of violaceous-to-hemorrhagic discoloration, focal forefoot ulceration, and marked diffuse induration (Figure 1).

**Figure 1:**
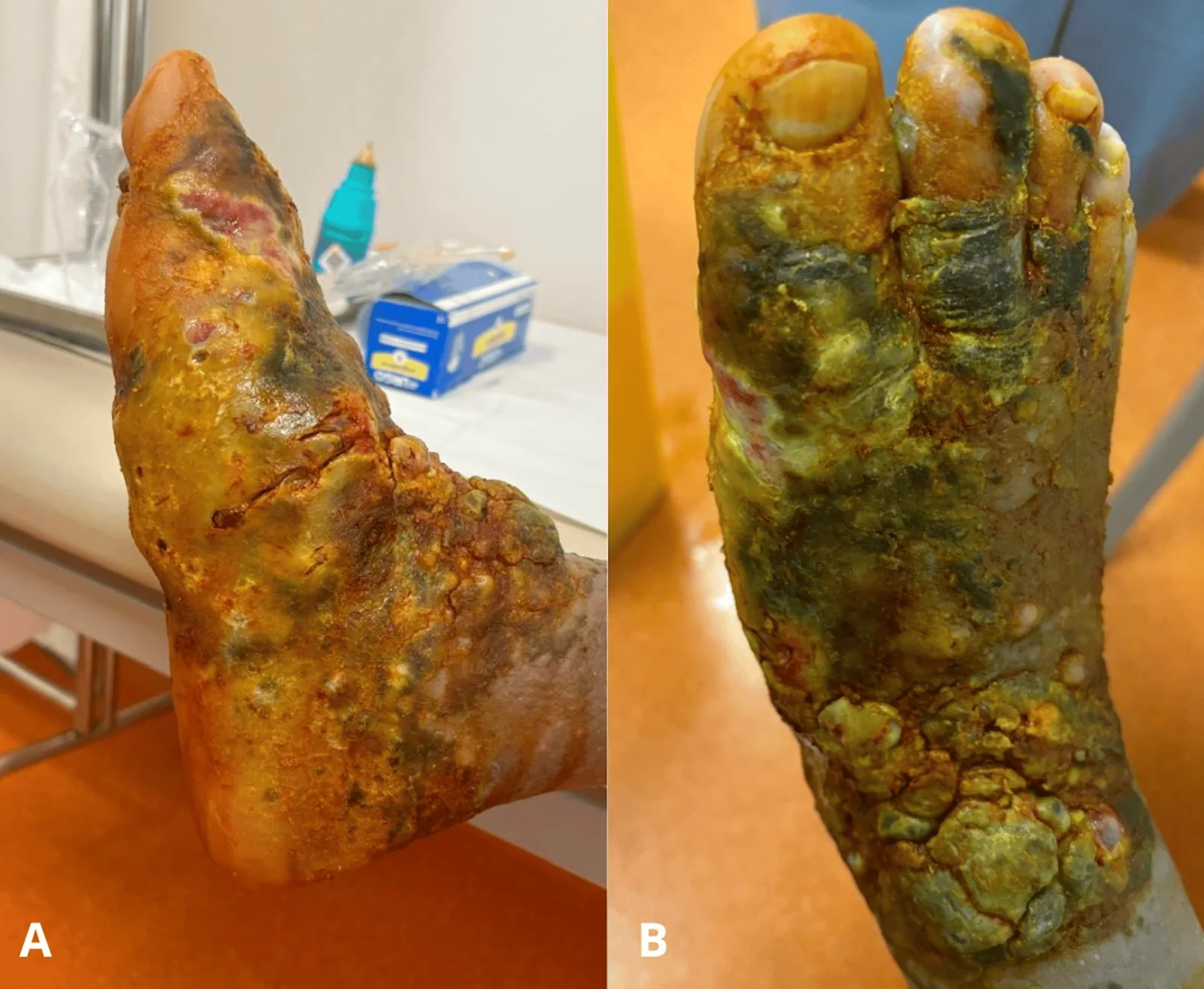
Clinical examination in July 2024. (A) Lateral view demonstrating confluent hyperkeratotic plaques and verrucous nodules with violaceous-to-hemorrhagic discoloration and focal ulceration. (B) Dorsal view showing extensive nodular lesions with hyperkeratotic crusting and diffuse induration of the right foot.

A skin biopsy was performed. Histopathological examination demonstrated a dermal spindle-cell nodular proliferation associated with intratumoral erythrocyte extravasation (Figure 2). Immunohistochemistry showed positive nuclear staining for HHV-8 latent nuclear antigen-1 (LANA-1), confirming the diagnosis of KS. HIV serology was negative, whereas HHV-8 serology was positive.

**Figure 2:**
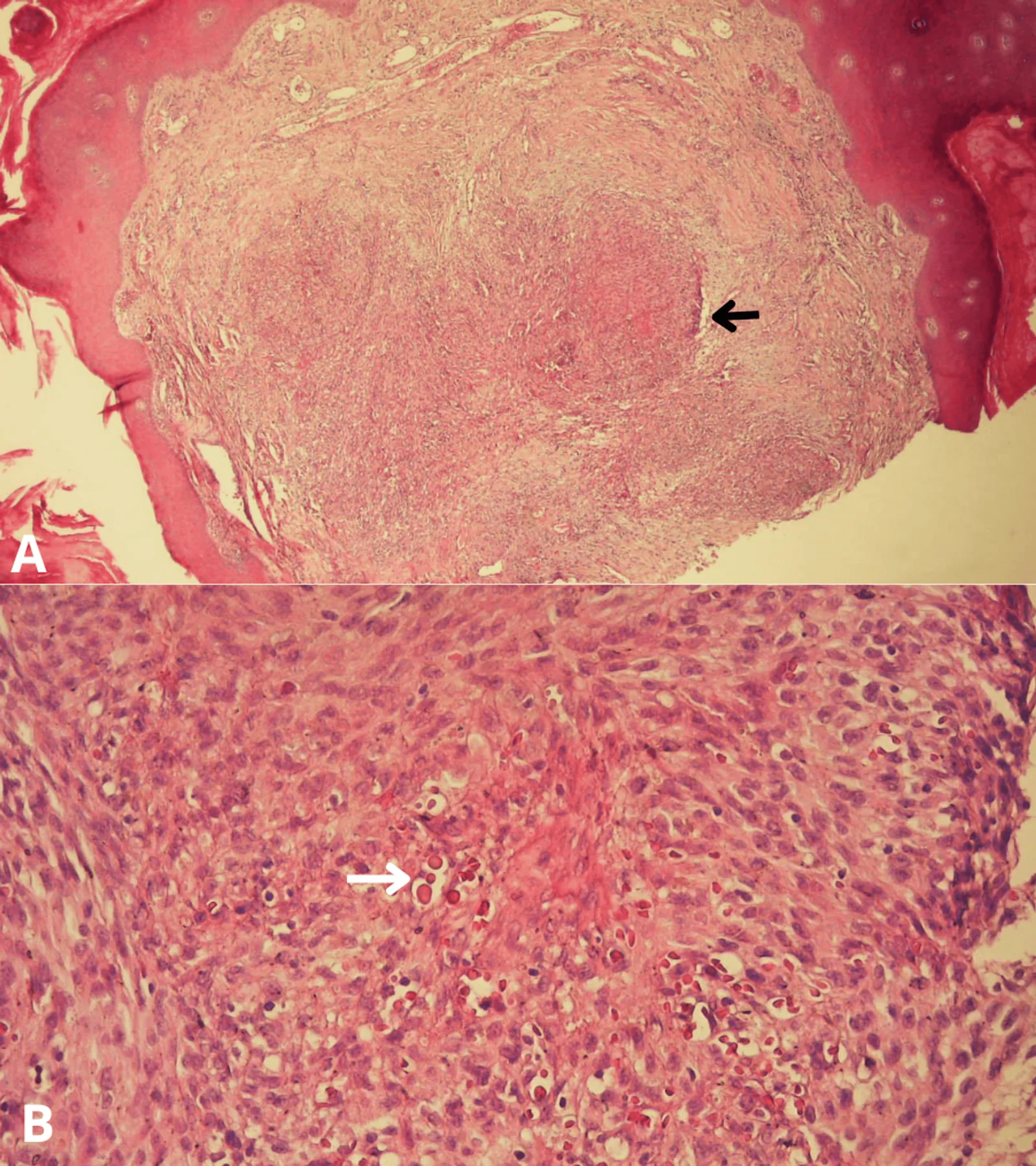
Histopathological findings. (A) H&E-stained section showing a dermal nodular proliferation of spindle cells (original magnification, ×2.5; black arrow). (B) Higher-magnification view showing erythrocyte extravasation within the spindle-cell proliferation (H&E; original magnification, ×10; white arrow).

The case was reviewed by a multidisciplinary tumor board involving hematologists, radiation oncologists, and dermatologists. Cutaneous involvement by lymphoma was initially considered the leading diagnosis, with KS regarded as an associated condition. However, a retrospective review of the patient’s clinical history and re-examination of the 2017 histological specimens by a pathologist, including HHV-8/LANA-1 immunostaining, confirmed that the first manifestations of KS had occurred as early as 2017. The disease had therefore remained unrecognized and had progressed insidiously for at least seven years before the correct diagnosis was established.

The patient subsequently received six cycles of rituximab plus bendamustine (R-BENDA) to treat the CLL/SLL; the treatment was well tolerated. Interim PET-CT evaluation demonstrated a partial metabolic response. Following completion of treatment, a repeat bone marrow biopsy showed a hypocellular marrow with maturation abnormalities and only a minimal reactive lymphocytic infiltrate on morphological and immunohistochemical examination, with no evidence of residual bone marrow involvement by CLL/SLL. Despite this hematological response, the KS lesions continued to progress. Paclitaxel-based chemotherapy was therefore initiated as second-line treatment specifically targeting KS. Six cycles, completed in April 2025, resulted in significant clinical improvement, with regression of the cutaneous lesions, improvement in the patient’s general condition, and restoration of ambulation.

In August 2025, two new nodules appeared on the right foot, suggesting disease progression, which was confirmed during follow-up evaluation in November 2025. At that stage, additional conventional chemotherapy was not considered feasible because of cumulative treatment-related toxicity, and no further systemic therapy was initiated.

The patient was subsequently referred to our dermatology department in December 2025 for further evaluation. His general condition remained relatively preserved. Dermatological examination revealed extensive confluent erythemato-violaceous nodules involving the plantar and dorsal surfaces of the right foot, consistent with KS (Figure 3). Dermoscopic examination demonstrated a characteristic rainbow pattern and serpentine vessels (Figure 4), findings consistent with the established dermoscopic features of KS.

**Figure 3:**
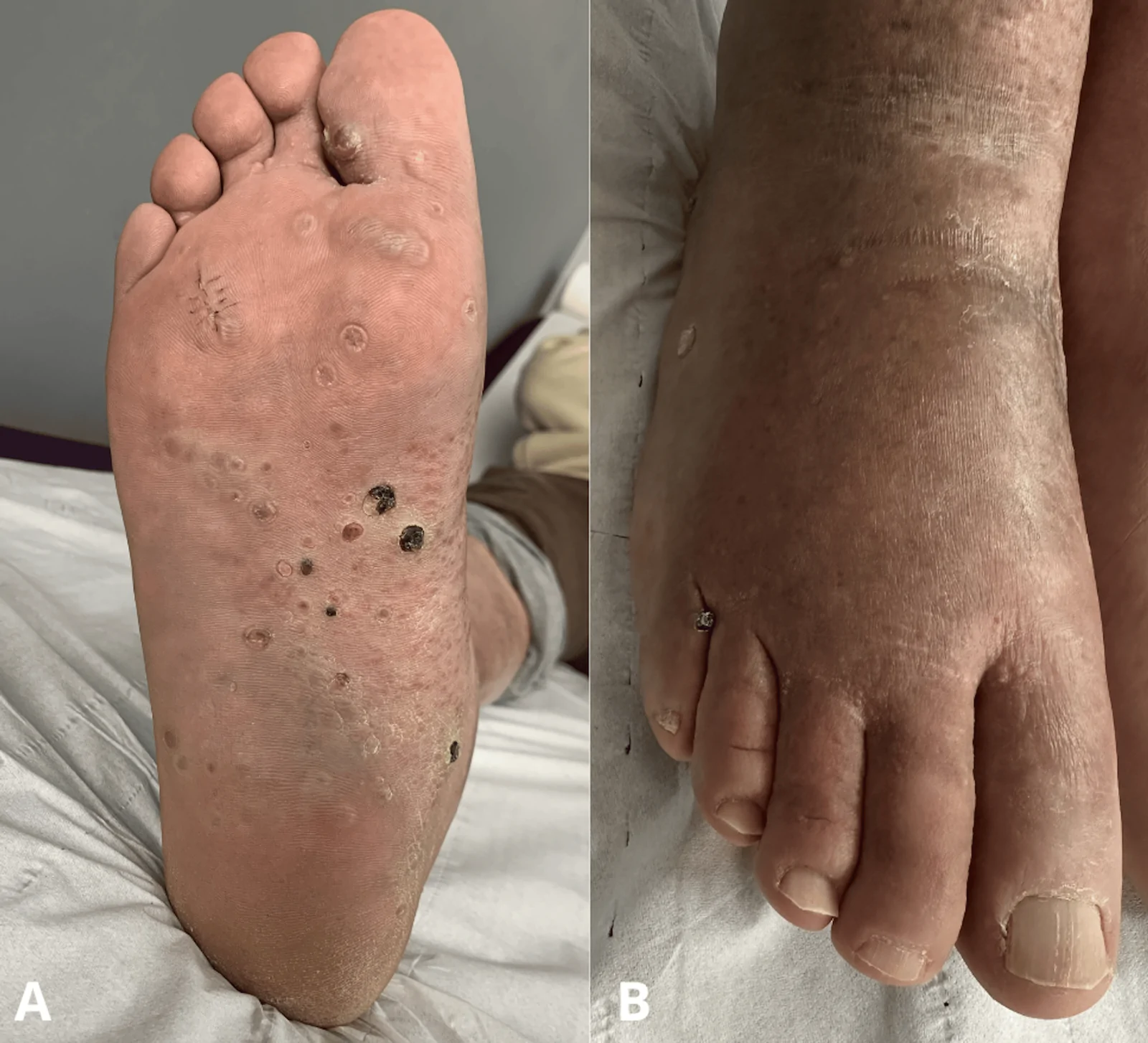
Clinical examination in December 2025. Extensive confluent erythemato-violaceous nodules involving the plantar (A) and dorsal (B) surfaces of the right foot, consistent with Kaposi sarcoma.

**Figure 4:**
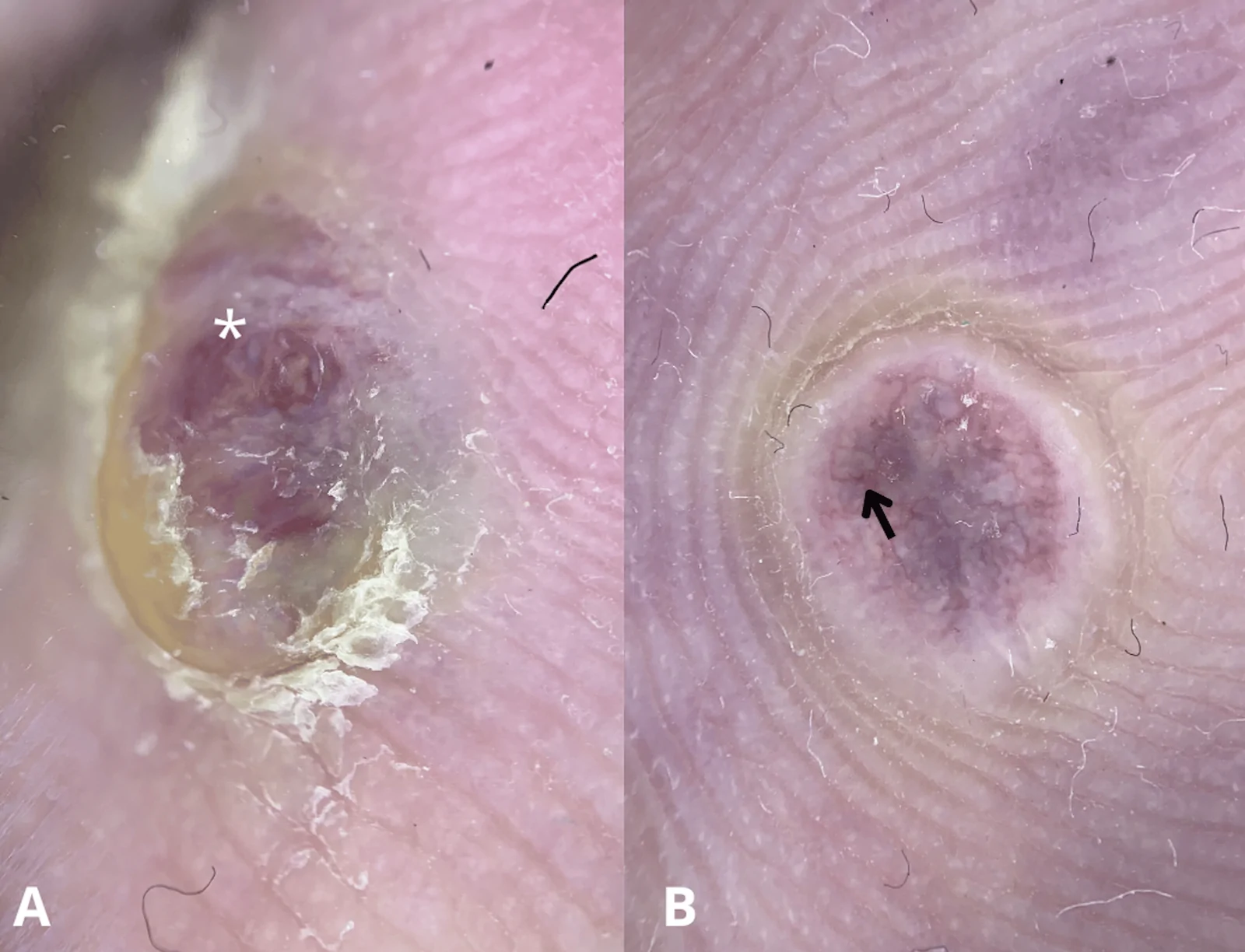
Dermoscopy of the plantar ulcerations. Dermoscopic images showing (A) a rainbow pattern (asterisk) and (B) serpentine vessels (arrow).

Given the absence of feasible systemic therapeutic options and the persistence of symptomatic nodular lesions, treatment with a long-pulsed Nd:YAG laser was undertaken. Two treatment sessions were performed, resulting in a favorable clinical response, with marked regression of the refractory nodules. The patient remains under close clinical follow-up.

## Discussion

The coexistence of KS and CLL/SLL in the absence of conventional immunodeficiency is exceptionally rare. Although HHV-8-associated lymphoproliferative disorders have been described, these disorders occur predominantly in the context of HIV infection or solid organ transplantation [5].

To our knowledge, KS preceding the diagnosis of CLL/SLL in an HIV-negative, non-transplant patient without prior immunosuppressive therapy represents an uncommon clinical presentation.

In our patient, retrospective re-examination of the 2017 histological slides confirmed that KS had been present for at least five years before the diagnosis of B-cell lymphocytic lymphoma in 2022. Interestingly, retrospective review showed that the complete blood count was normal in 2017, whereas a CBC performed in 2019, three years before the diagnosis, revealed leukocytosis (12,000/mm³) with absolute lymphocytosis (8,227/mm³). These findings were attributed at the time to a presumed infectious cause and were not further investigated using flow cytometry. Although clonality could not be retrospectively confirmed, this finding raises the possibility that a monoclonal B-cell population was already present several years before the formal diagnosis of CLL/SLL. This chronological sequence suggests that KS may precede the clinical recognition of CLL/SLL and raises the possibility that subclinical immune dysregulation related to an evolving, clinically silent B-cell clone may have contributed to HHV-8-driven tumorigenesis long before the hematological diagnosis was established. This hypothesis is consistent with the growing recognition that monoclonal B-cell lymphocytosis (MBL) can represent a clinically and biologically silent precursor state, sometimes persisting for years before progressing to overt CLL/SLL. As recently emphasized by Serafin A et al., high-count MBL shares genetic and biological features with early-stage CLL and is associated with measurable immune dysfunction even before the formal diagnostic threshold for CLL is met. This underscores the importance of not overlooking subtle, early hematological abnormalities such as the one retrospectively identified in our patient [6].

The prolonged interval between the initial lesions and the definitive diagnosis likely reflects the indolent and atypical clinical presentation of KS in this patient. Although not the central feature of the case, this observation highlights the value of repeat biopsy and histopathological review when chronic cutaneous lesions remain unexplained.

Whether HHV-8 represents a shared oncogenic driver or promotes lymphoproliferation within a shared immunological environment remains uncertain. HHV-8 can infect B lymphocytes and is implicated in the development of several lymphoproliferative disorders [7]. Although direct causality in CLL/SLL remains to be established, the biological capacity of HHV-8 to drive lymphoproliferation provides a plausible mechanism linking the two neoplastic processes in this patient.

The absence of HIV infection, organ transplantation, or corticosteroid therapy in our patient raises the question of how HHV-8 reactivation was permitted. CLL/SLL is well established as a cause of functional immunosuppression, even in the absence of treatment [4]. The immunosuppressive microenvironment driven by leukemic B cells in patients with CLL/SLL, the downregulation of CD40 ligand expression on activated T cells, and altered granulocyte and T-cell function all contribute to immune impairment, leading to an increased incidence of secondary malignancies [8]. More specifically, one hypothesis linking CLL/SLL to KS is based on the role of an ineffective T-cell response to latent HHV-8 infection, as the intrinsic immunosuppression associated with CLL/SLL may predispose to viral reactivation through an imbalance in T-cell subpopulations [4]. Furthermore, HHV-8 infection alone is insufficient to cause KS; a combination of viral infection, a compromised immune system, and oncogenic mechanisms is required for tumorigenesis [9]. In our patient, CLL/SLL-associated immune dysfunction, even without formal immunodeficiency, may have provided precisely this permissive environment.

The management of concurrent KS and CLL/SLL in an HIV-negative, non-transplant setting is not standardized, and no established guidelines specifically address this combination.

Although R-BENDA achieved a partial response in the underlying lymphoma, KS continued to progress during chemotherapy. This discordance between lymphoma response and KS progression is noteworthy and raises the possibility of an iatrogenic effect of rituximab on HHV-8-driven disease. This observation is consistent with reports of rituximab-induced KS exacerbation in HIV-negative patients, in whom B-cell depletion may paradoxically impair immune control of HHV-8 [10,11].

Following the incomplete lymphoma response and concurrent progression of the KS lesions, paclitaxel was subsequently introduced by the multidisciplinary tumor board as a pragmatic second-line option, primarily targeting the refractory KS component. This approach is consistent with its established efficacy in advanced KS, with reported response rates of 56%-71% [12]. Six cycles yielded meaningful clinical improvement, including restoration of ambulation, before subsequent disease progression. After two lines of chemotherapy, no further conventional treatment was considered feasible because of cumulative toxicity.

In addition, two sessions of long-pulsed Nd:YAG laser treatment were performed for the persistent nodular lesions, resulting in a favorable local response. Although evidence remains limited, vascular lasers may represent a useful adjunctive option for localized, refractory KS lesions, particularly when systemic therapeutic alternatives are limited or exhausted [13].

Emerging data also suggest a potential role for immune checkpoint inhibitors in advanced or refractory KS. Although the evidence remains limited to small early-phase trials, case series, and case reports, durable responses have been reported, making this approach a promising avenue for further investigation [14,15].

## Conclusions

This case illustrates the coexistence of classic KS and CLL/SLL in an HIV-negative, non-transplant patient. The retrospective identification of KS in histological specimens obtained seven years before the definitive diagnosis highlights the potential for prolonged diagnostic delay and emphasizes the value of histopathological re-evaluation of atypical or persistent cutaneous lesions. CLL/SLL-associated immune dysfunction may have provided a permissive environment for HHV-8-driven oncogenesis, although this relationship remains hypothetical. Finally, this case underscores the therapeutic challenges posed by KS in patients with lymphoproliferative disorders, as treatment directed at one disease may exacerbate the other. In selected cases, local modalities such as Nd:YAG laser therapy may represent a useful adjunctive option when systemic treatment becomes ineffective or poorly tolerated.
